# Deep Learning Methods for Predicting Disease Status Using Genomic Data

**Published:** 2018-12-11

**Authors:** Qianfan Wu, Adel Boueiz, Alican Bozkurt, Arya Masoomi, Allan Wang, Dawn L DeMeo, Scott T Weiss, Weiliang Qiu

**Affiliations:** 1 Questrom School of Business, Boston University, 595 Commonwealth Avenue, Boston, MA, 02215, USA; 2 Channing Division of Network Medicine, Brigham and Women’s Hospital/Harvard Medical School, 181 Longwood Avenue, Boston MA 02115, USA; 3 Department of Medicine, Pulmonary and Critical Care Division, Brigham and Women’s Hospital, Harvard Medical School, Boston, MA, USA; 4 Department of Computer Science, Northeastern University, Boston, MA, USA; 5 Belmont High School, Boston, MA, USA

**Keywords:** Deep learning, Auto-encoders, Genomic data, Disease prediction, Dimension reduction

## Abstract

Predicting disease status for a complex human disease using genomic data is an important, yet challenging, step in personalized medicine. Among many challenges, the so-called curse of dimensionality problem results in unsatisfied performances of many state-of-art machine learning algorithms. A major recent advance in machine learning is the rapid development of deep learning algorithms that can efficiently extract meaningful features from high-dimensional and complex datasets through a stacked and hierarchical learning process. Deep learning has shown breakthrough performance in several areas including image recognition, natural language processing, and speech recognition. However, the performance of deep learning in predicting disease status using genomic datasets is still not well studied. In this article, we performed a review on the four relevant articles that we found through our thorough literature search. All four articles first used auto-encoders to project high-dimensional genomic data to a low dimensional space and then applied the state-of-the-art machine learning algorithms to predict disease status based on the low-dimensional representations. These deep learning approaches outperformed existing prediction methods, such as prediction based on transcript-wise screening and prediction based on principal component analysis. The limitations of the current deep learning approach and possible improvements were also discussed.

## Introduction

Complex human diseases, such as cancers, cardiovascular diseases, and respiratory diseases, have caused huge public health concerns and economic burdens [1,2]. It is believed that both environmental factors, such as smoking exposure, nutrient intake, physical exercise, and genomic factors contribute to the development of complex human diseases [3]. We refer genomic factors to any molecular factors related to genes, such as genotype, mRNA expression, DNA methylation, microRNA expression, metabolites, proteins, etc. Cutting-edge technologies, e.g., genotyping and next-generation whole genome sequencing, greatly facilitate the investigations of the associations of genomic factors to complex human diseases so that researchers can unbiasedly detect disease-associated factors. In addition to uncovering the underlying molecular mechanisms, researchers expect that the disease-associated genomic factors could also help diagnose disease, personalize treatment, and develop new medicines [4].

**Figure 1:**
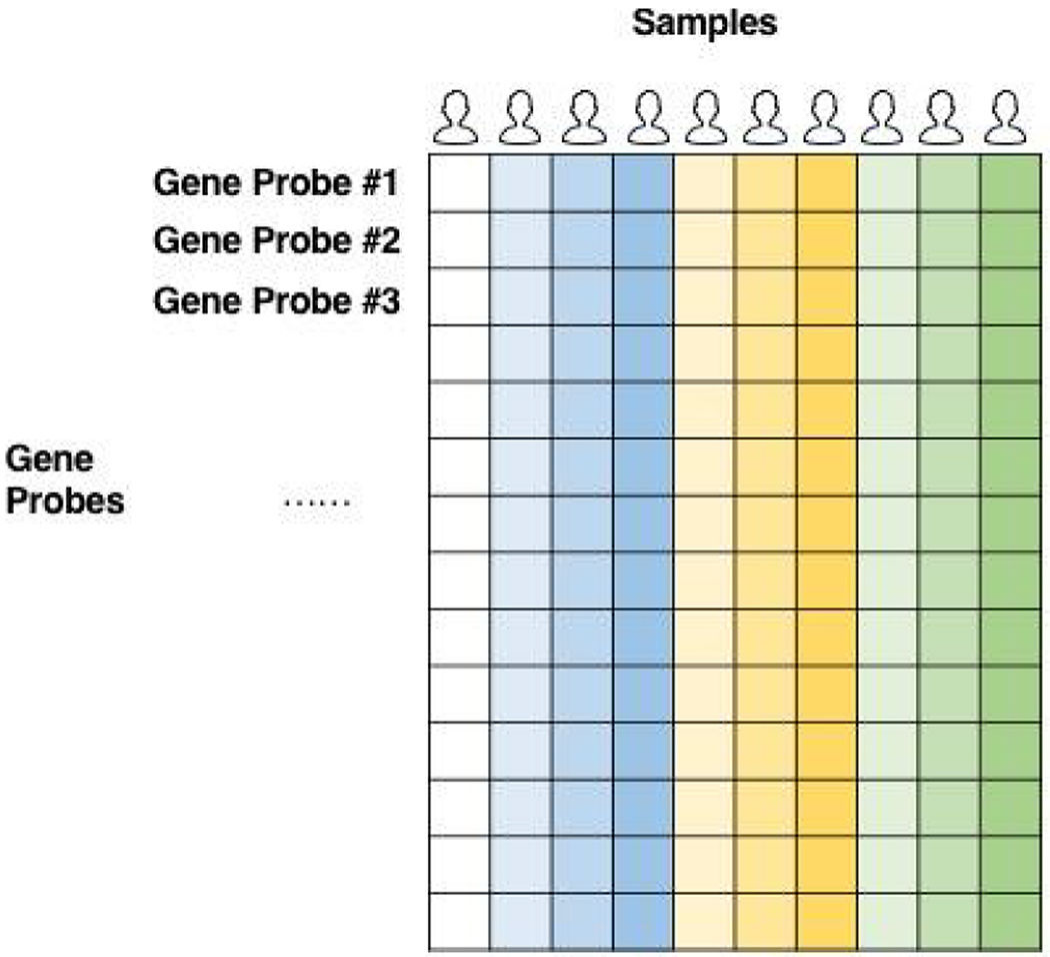
An illustration of gene expression data. In the above figure, each row represents 1 gene transcript and each column represents one sample (one person). The (i,j) cell records the expression level of the i-th gene transcript for the j-th sample. Gene expression data typically have high dimensionality (20,000-50,000 gene transcripts) and small sample size (<1000), resulting in the “curse of dimensionality problem”.

Several machine learning methods, such as support vector machine [5] (SVM), random forest [6], and k-nearest neighbors [7] have been successfully applied in disease prediction based on clinical data [8– 10]. For genomic data generated by high-throughput technologies (Figure 1), the major challenge in disease prediction is the “curse of dimensionality” [11–13], which refers to the scenario where the number of genomic factors is far larger than the number of samples, resulting in model over-fitting and computational inefficiency. Model over-fitting is the phenomenon that a model fits a particular set of data too closely or exactly to fitting additional data or future observations reliably.

**Figure 2:**
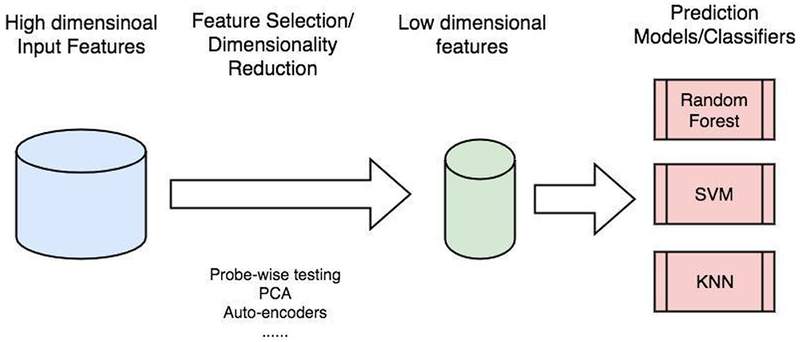
An illustration of building prediction models using genomic datasets. The idea is to first reduce the dimensionality of the input features and then feed the low dimensionality features into prediction model/classifiers. Dimensionality reduction techniques typically include transcript-wise testing, principal component analysis (PCA), and auto-encoders.

A reasonable approach [14,15] to handle the curse of dimensionality is to first apply feature selection techniques to select key features relevant to the disease of interest, and then to predict the disease status based on these key features (Figure 2). In genomic data analysis, a feature can be a gene transcript or a (non) linear combination of several gene transcripts. Traditional feature selection techniques include forward variable selection, backward variable deletion, stepwise variable selection, transcript-wise tests, or principal component analysis. These methods have limited performance in genomic data analysis. Forward variable selection, backward variable deletion, and stepwise variable selection are time-consuming. Hence, they are not suitable for whole genome-wide analysis. Transcript-wise tests ignore the fact that many omics variables are correlated and therefore carry redundant information regarding prediction. Ignoring the redundancy would result in the selected transcripts are non-reproducible in independent cohorts [13,16,17]. In addition, contributions of different genomic risk factors might vary; however, transcript-wise tests implicitly assign equal weights to all selected transcripts. Principal component analysis (PCA) explicitly assigns different weights to different transcripts. However, PCA produces a linear combination of transcripts and ignores the possible non-linear relationship between transcripts.

Recently, deep learning methods have made breakthrough progress in image/video recognition [18], natural language processing [19], and robotics [20,21]. Through a stacked and hierarchical learning system, deep learning methods could efficiently capture complex relationships between high-dimensional features, either spatial or consequential [22].

In bioinformatics, deep learning methods have fruitful and innovative applications in medical image classification [23,24], predicting DNA- and RNA-binding proteins sequences [25], and DNA sequence noncoding variants effects predicting [26]. However, using deep learning methods to predict disease status is not a well-researched area.

Many investigators in genomic data analysis fields might hear about deep learning and would like to learn more about it and how it could be used to predict disease status based on genomic data. In this review, we will first introduce the main components of deep learning and the most frequently used deep learning feature extraction methods in genomic data analysis. We will then review the papers that used deep learning to predict complex human diseases based on genomic data. The limitations of the current deep learning approach and possible improvements will also be discussed.

## Survey Methodology

To thoroughly search recent literature on deep learning applications in disease prediction, we carefully reviewed previous works, searched popular sites: Google Scholar, PubMed, IEEE Xplore, and PMC, and examined related online blogs and tutorials, such as GitHub (http://github.com/), Kaggle (http://www.kaggle.com/), and Cross Validated (https://stats.stackexchange.com/). We identified four papers [13,27-29] published between January 2013 and December 2017, which applied deep learning methods in disease prediction using genomic data.

Before we review the details of the four studies, we first introduce in the following sections the main components of deep learning and the most frequently used deep learning feature extraction methods in genomic data analysis.;

## Neural Networks (ANNs) and Deep Learning Methods in Predicting Disease

The main component of all deep learning algorithms is Artificial Neural Networks (ANNs). Understanding how ANNs are constructed and trained is the first step to understand deep learning methods.

### Artificial neural networks (ANNs)

**Figure 3:**
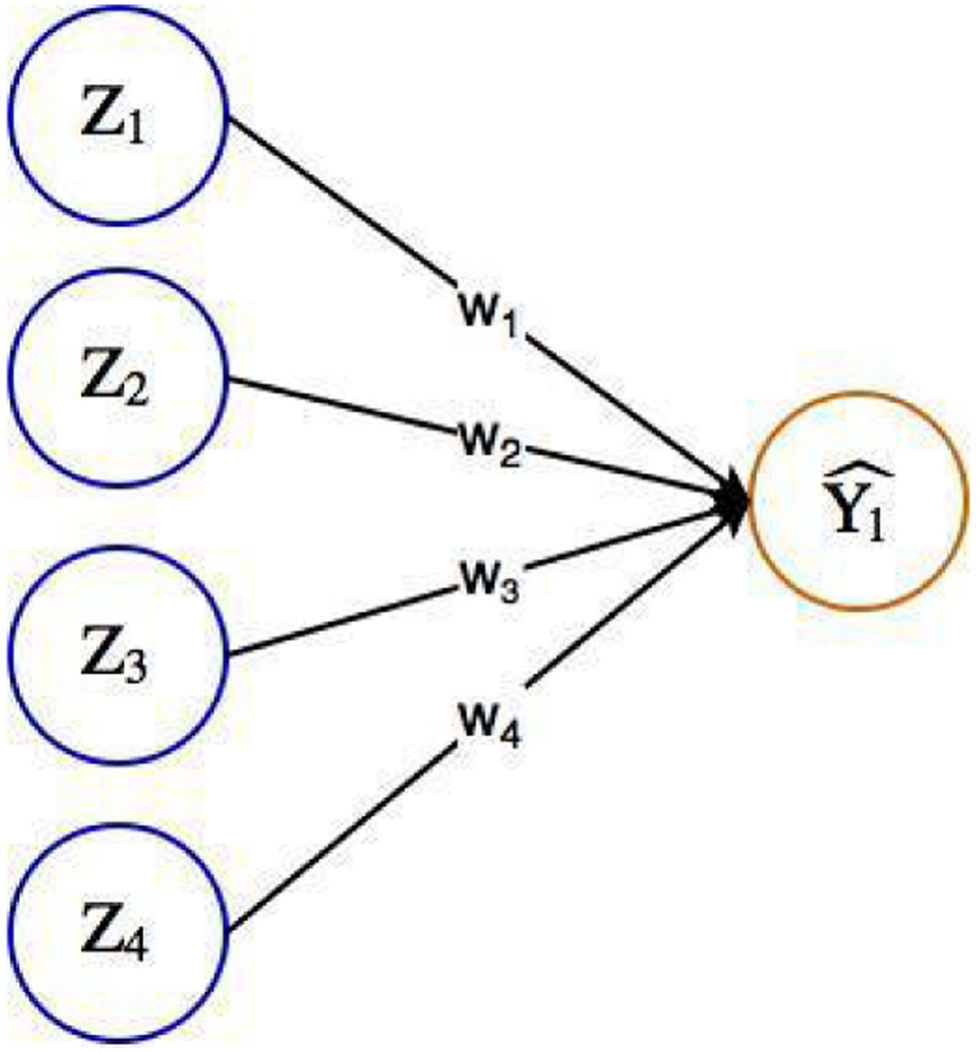
An illustration of a simple ANN: This simple feed-forward ANN has four input nodes and one output node. On the edges, w_1_–w_4_ represent the weights of the input nodes. The value Y_1_for the output node is computed as ${\hat{Y}}_{1}=f(b+{Z_{1}}^{*}w_{1}+{Z_{2}}^{*}w_{2}+{Z_{3}}^{*}w_{3}+{Z_{4}}^{*}w_{4})$, where b is the bias term, and *f* is the activation function.

**Figure 4:**
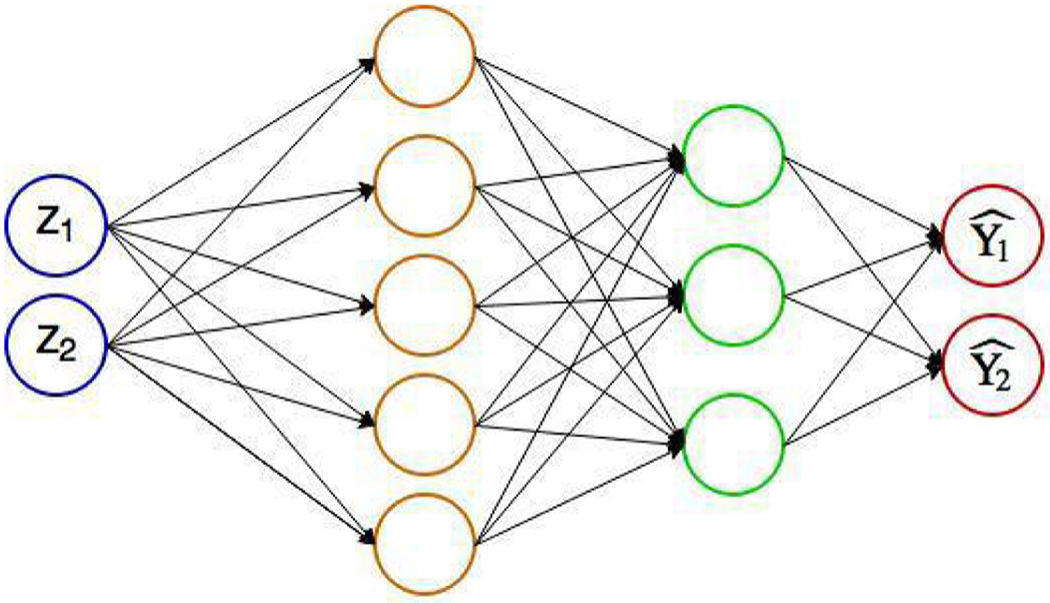
An illustration of a multiple-layer ANN. This multiple-layer ANN has one input layer, two hidden layers, and one output layer, with each layer connected to the previous layer. The activation function *f* is applied to each node on the hidden layer and the output layer.

Artificial neural networks are computing systems that are inspired by the biological neural networks constituting brains. Typically, an ANN is a network of nodes with multilayers: one input layer, one output layer, and several hidden internal layers. Within a layer, nodes are not connected, while between the layers nodes are fully connected (Figures 3 and 4). Each node can store a value. For instance, in Figure 3 *Z_i_* is the value stored in the *i*-th node. Each edge can have a weight. For example, the weight *w_ji_* indicates the amount of information passing to the node *i* in the given layer from the node *j* in the previous layer. The value of a node on a given layer, except for the input layer, is a function of a bias (i.e., threshold; e.g., *b_i_* for the *i*-th node) and the weighted average values of all nodes on the previous layer. The function is called an activation function. For instance, ${\hat{Y}}_{1}=1$ *if*(*b_*i*_* + *w*_1*i*_ * *Z*_1_ + … + *w_ni_* * *Z_n_*) > 0 and ${\hat{Y}}_{1}=0$ otherwise, where *n* is the number of nodes in the previous layer and *Z_j_* is the value for the *j*-th node in the previous layer. Usually, activation functions, such as sigmoid, rectified linear unit (ReEU) [30], and hyperbolic tangent (Tanh), are non-linear.

### Training ANNs

To estimate the optimal values of the biases and edge weights, a training data set and a validation set are needed, in which the values of the nodes in the output layers are known. The idea is to find a set of biases and edge weights that minimize the difference between the true values and predicted values of nodes in the output layer. The difference is a function of the biases and edge weights and is usually called loss function.

**Figure 5:**
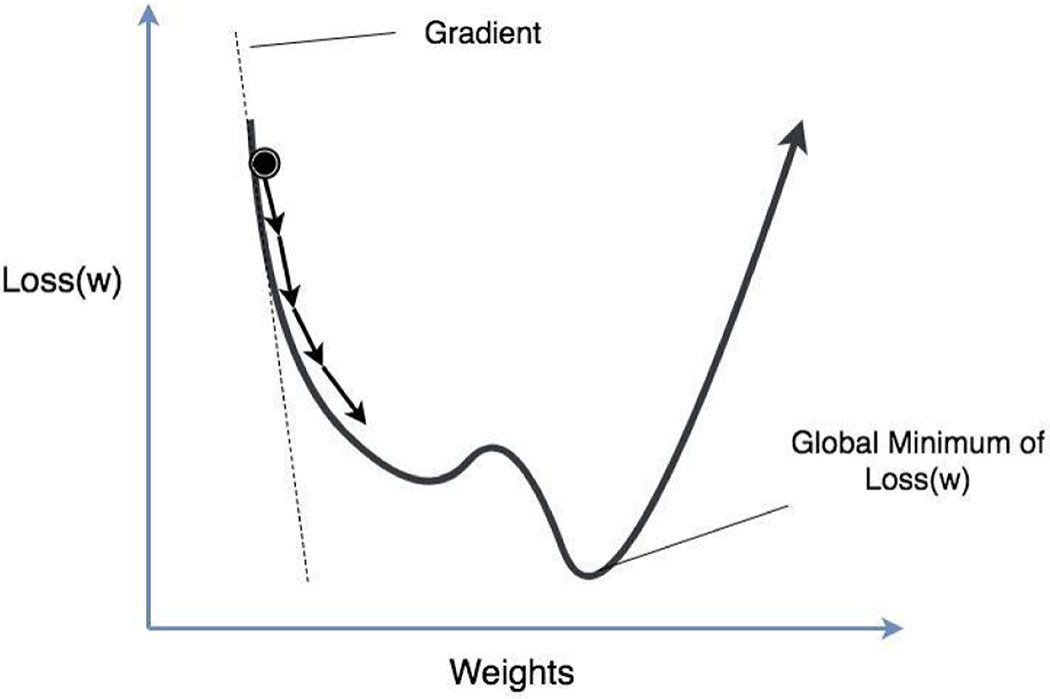
Gradient Descent Training. The x-axis is the weight w and the y-axis is the loss function Loss (w). In Gradient Descent optimization, learning rate represents how much the edge weights are adjusted in each step before the global minimum is achieved. Learning rate could also be seen as the “step size” in the learning process. With a higher learning rate, the gradients are adjusted by a greater amount each step. With a lower learning rate, the gradients are adjusted by a smaller amount each step.

Gradient descent is an optimization method for updating the parameters of a neural network to minimize the loss function (Figure 5). It uses the fact that optimal parameters are achieved when the gradient of the loss function with respect to the parameters are zero. However, finding parameters that are the solution to zero gradient equation is a nontrivial task for complex networks with a large number of parameters. An alternative method to solving the gradient equation is, starting with an initial point, to iteratively update each parameter proportional to the negative of the gradient of the loss function with respect to the parameter, and continue this procedure until the amount of change of parameters is below a predefined threshold. An important part of this method is to calculate the gradient of loss function with respect to every parameter in the network. Backpropagation is an algorithm for efficiently calculating the gradient for each parameter, using the chain rule: For the simple network in Figure 3, $\frac{\partial \mathrm{Loss}(w)}{\partial w_{1}}=\frac{\partial \mathrm{Loss}(w)}{\partial {\hat{Y}}_{1}}\frac{\partial {\hat{Y}}_{1}}{\partial w_{1}}$, where *Loss*(*w*) is the loss function. This implies that once we know the gradients at some layer, we can easily calculate the gradients for the layer before it.

### Deep learning and deep neural networks (DNNs)

ANNs with only one ortwo hidden layershave a shallow architecture, which contains only two levels of data-dependent computational elements and can be very inefficient regarding the number of hidden nodes, and in terms of required training examples [11]. In contrast, deep neural networks are ANNs with more than two hidden layers. This deep architecture can compactly represent a large number of computational elements via the composition of many nonlinearities [11]. Deep learning methods are defined as computational models that are composed of multiple processing layers to learn representations of data with multiple levels of abstraction [22].

The performance of deep learning relies on the methods to train the parameters in DNNs. Intuitively, we can train the parameters by minimizing the prediction error rates (the loss function) by applying gradient descent. However, empirical experiments showed that this supervised approach has poor performance for DNNs [11,31], in the regime where the number of input features is comparable to (or even far larger than) the number of training samples, which is the case in genomic datasets. In contrast, unsupervised learning at each stage of a deep network proposed by the seminal works of Hinton et al. [32] and Hinton and Salakhutdinov [33] pretrains each hidden layer as the encoder of an auto-encoder trying to reconstruct the output of the previous layer. Hence, combining unsupervised approach with the supervised approach, such as fine-tuning all the parameters of the ANN using backpropagation and gradient descent on a global supervised cost function, can significantly improve the performance of deep learning methods for data-sparse datasets [11,31].

### Auto-encoder (AE)

**Figure 6:**
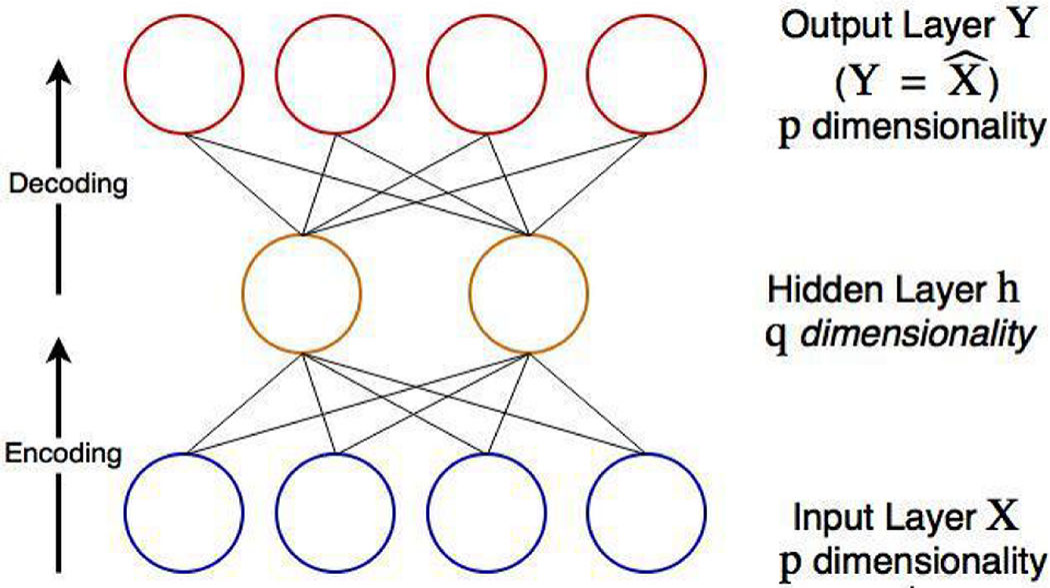
Illustration of a basic auto-encoder. This auto-encoder has 2 hidden units. **X** is the inputs, **Y** = $\hat{X}$ is the reconstructed inputs in the output layer, h is the hidden layer. The dimension of the original input data is reduced from p=4 to q=2. The optimal representation in the q-dimensional space is obtained by minimizing the difference between the inputs **X** and the reconstructed inputs **Y**

An auto-encoder is a type of ANN that aims to find a new representation of input nodes (e.g., gene transcripts in genomic data analysis) in an *unsupervised* manner, from which the input can be reconstructed without too much loss of information [31]. Like ANN, an auto-encoder has one input layer, one output layer, and one or multiple hidden layers (Figure 6). Suppose *X* is the original data in a *p*-dimensional space. An auto-encoder would first project ***X*** to a *q*-dimensional space ***Y**=g_1_*(***X***), where *g_1_* is a non-linear projection function. Then it transforms ***Y*** back to the *p*-dimensional space ***Z**=g_2_(**Y**)*, where *g_2_* is also a non-linear projection function. The optimal projection Y* minimizes the loss function *loss[**X**, g_2_(**Y**)]* that measures the differences between ***X*** and ***Z**=g_2_ (**Y**).* Note that since *q* is different from *p*, both the projection function *g_1_* and the projection function *g_2_* are not one-to-one mapping functions. Hence, the inverse functions *g_1_^−1^* and *g_2_^−1^* do not exist.

Similar to training ANNs, backpropagation and gradient descent can be applied to train an auto-encoder, in which the output layer has the dimension as the original data ***Z**=g_2_(**Y**)=g_2_(g_1_(**X**))*.

The nodes ***Y**=g_1_(**X**)* within the hidden layer are the representations of original features. The hidden layer is “under-complete” if the number (*q*) of nodes in the hidden layer is smaller than that (*p*) in the input layer (*q*<*p*). In most cases, auto-encoder outperforms Principal Component Analysis in processing high dimensional complex datasets because auto-encoder performs both linear and non-linear projections, while PCA performs only linear projection. Auto-encoders have been successfully used to efficiently extract meaningful features in disease diagnosis based on high-throughput genomic data [27,34].

### Sparse auto-encoder (SpAE)

**Figure 7:**
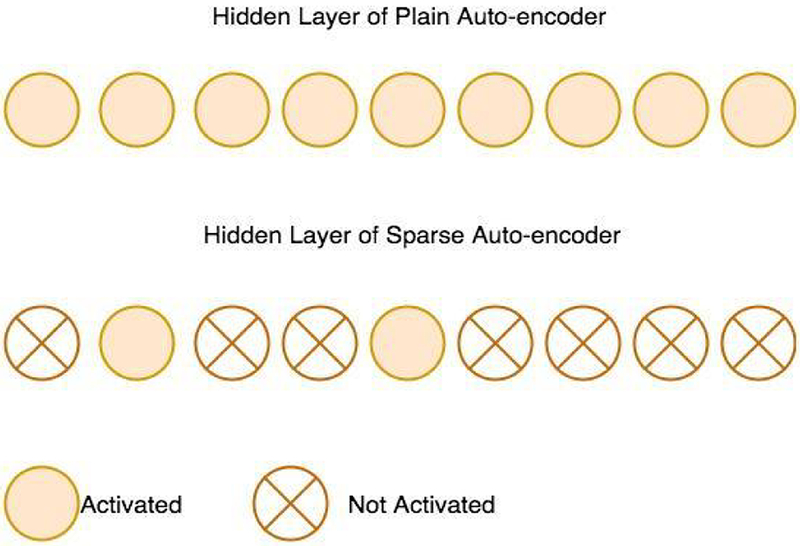
Illustration of a sparse auto-encoder: A sparse auto-encoder restricts the number of hidden layers activated by adding a sparsity term to the loss function. The sparsity term set the expected activation value of the hidden nodes to a small constant so that most of the hidden nodes’ activations are near zero. Hence, very few hidden nodes are activated in a sparse auto-encoder.

Performing backpropagation and gradient descent could be inefficient if there are too many free nodes with complex dependencies in each layer [35,36]. Sparse auto-encoder is developed to restrict the number of hidden nodes to be activated by introducing sparsity-constraints on the hidden units (Figure 7). Sparse auto-encoder have been proved to have favorable performance in image recognition [37] and speech emotion recognition [38], due to its efficiency in extracting meaningful features from high-dimensional data.

### Stacked auto-encoder (StAE)

**Figure 8:**
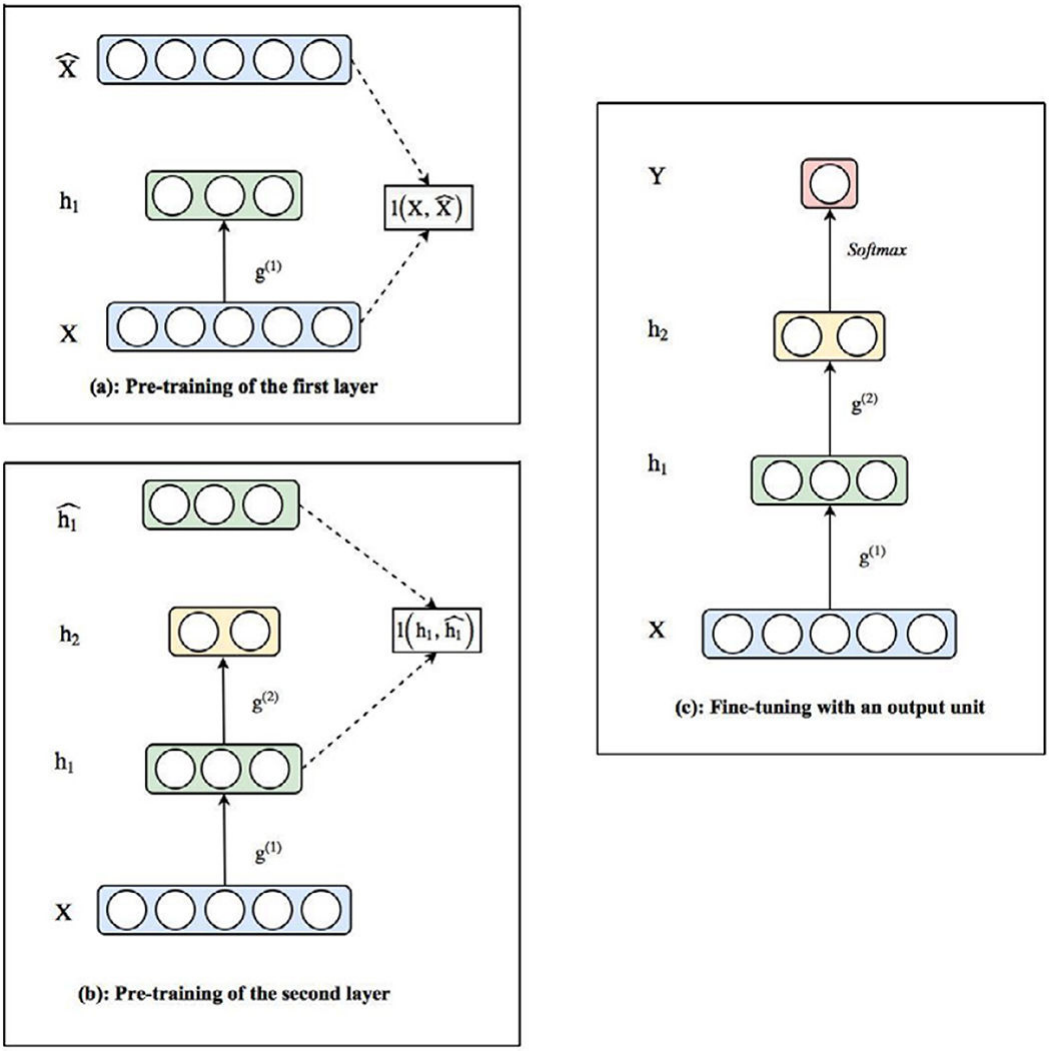
Illustration of stacked auto-encoder and greedy layer-wise pre-training: The stacked auto-encoder has 2 hidden layers h_1_ and h_2_. Under the greedy layer-wise pre-training, hidden layer h_1_ is first trained in the same way as training a simple 1-layer auto-encoder by minimizing $1(X,\hat{X})$. The function g^(1)^ that maps X to h_1_ is learned from the first layer training, which is shown in (a). Then nodes values on h_1_ are passed to the second layer h_2_ to train the function g^(2)^ that maps h_1_ to h_2_ by minimizing $1(h_{1},{\hat{h}}_{1})$, which is shown in (b). After pre-training all hidden layers, an output unit Y, which serves as a classifier, could be wired on top of the hidden layers to make predictions. The whole architecture could be fine-tuned together using backpropagation and labeled data, which is shown in (c).

A stacked auto-encoder [11,39,40] is a multi-layer auto-encoder, each hidden layer of which is a representation of the previous layer obtainedby an auto-encoder with one hidden layer (Figure 8). The training of stacked auto-encoders is often completed by applying the greedy layer-wise pre-training approach [11]. Given extremely high-dimensional input data, a stacked auto-encoder could extract features layer by layer and finally forms a better representation to be passed into classifiers.

### Denoising auto-encoder (DAE)

**Figure 9:**
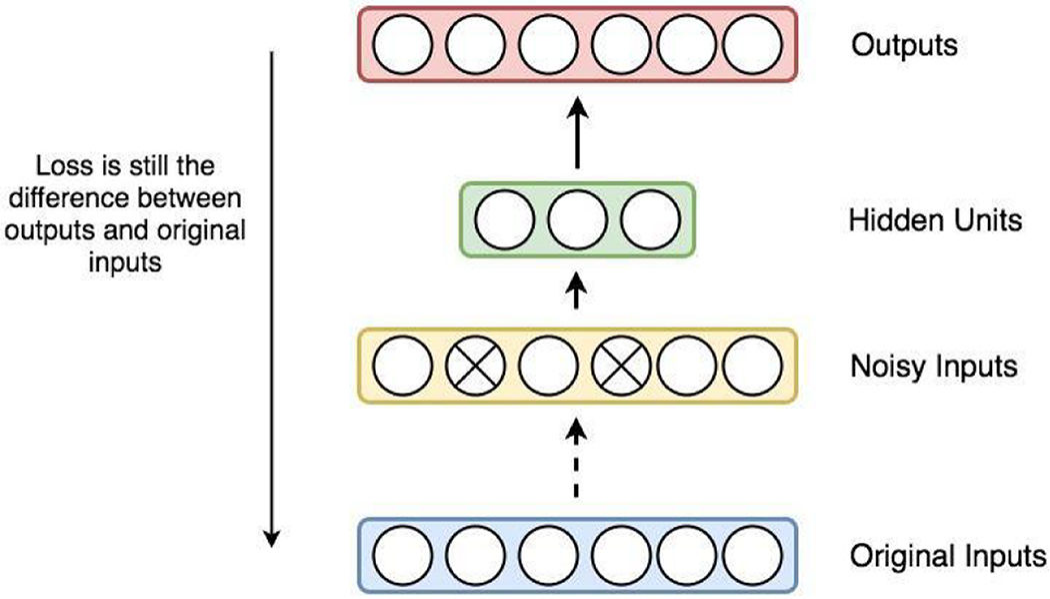
Illustration of a denoising auto-encoder. A denoising auto-encoder first transforms original inputs into noisy inputs. However, the loss in each step of the training process is still computed by the difference between the reconstructed representations in the output layer and the original inputs in the input layer.

A basic auto-encoder could successfully retain much of the information from the inputs in new features within the hidden layer. However, Vincent et al. [40] demonstrated that simply retaining information from the inputs does not guarantee that the extracted features are “good features”, which could achieve high-performance in supervised learning tasks. Denoising auto-encoder has been proposed to overcome this challenge by generating a noisy representation based on the inputs, such as setting values to 0 for a small proportion of input nodes or adding a noise term with a Gaussian distribution, and then feeding the noisy term into the auto-encoder (Figure 9). With the introduction of the noise term to the original inputs, denoising auto-encoders construct more robust feature representations and thereby could generalize better to unseen examples and datasets.

### Stacked denoising auto-encoder (SDAE) and stacked sparse auto-encoder (SSAE)

An SDAE is a multi-layer auto-encoder, each hidden layer of which is a representation of the previous layer obtained by a denoising auto-encoder with one hidden layer. For example, when pre-train the 2 hidden layers *h*_1_ and *h*_2_ in Figure 8, one could add a noise term to the pre-training inputs *X* and *h*_1_ to construct SDAE. Vincent et al. [40] showed that the features extracted by SDAE are stable and robust under noisy inputs, by achieving the best classification results under 9 out of 10 image databases. These features could efficiently capture useful information in the input distribution and have yield equivalent or better classification performance over most of the image data processing benchmarks. Similar to SDAE, an SSAE is obtained when the number of hidden units to be activated is restricted on each hidden layer of a stacked auto-encoder. Xu et al. [41] applied SSAE on Breast Cancer detection using image data. The study shows that SSAE outperformed 9 other state of the art cancer detection strategies and improved F-measure to 84.49%.

## Deep Learning Applications in Disease Prediction

### Previous works of disease prediction in genomic data Analysis using non-deep learning approach

Plenty of methods have been proposed in disease prediction using genomic data (e.g., [42–47]). Due to the large number of predictors (i.e., gene transcripts), the main approach in disease detection/prediction is to first obtain a subset of gene transcripts (e.g., a few top gene transcripts in transcript-wise tests) ora subset of representations of gene transcripts (e.g., a few top principal components), and then to predict disease status based on the selected transcripts or representations using machine learning algorithms.

Furey et al. [42] used SVMs to classify cancer tissue samples using gene expression datasets. The study showed that SVMs are able to classify tissue and cell types based on gene expression data and have similar performances to other machine learning methods. Khan et al. [43] was among the first to adopt basic ANNs (ANNs without hidden layers) to classify cancer samples and to identify relevant genes. In their study, the 10 top PCA components were used as inputs to the ANN to classify the small, round blue-cell tumors (SRBCT) to four distinct diagnostic categories. All 63 samples in the training set and all 25 samples in the independent testing set were correctly classified based on the 96 selected genes. Pal et al. [44] proposed to combine modified perceptron network and relational fuzzy clustering algorithms [48] to select a gene subset used for cancer subgroup classification. They applied their method to the SRBCT dataset analyzed by Khan et al. [43] and identified 7 genes that can accurately classify the samples in both training set and testing set. Chang et al. [45] used an ANN with one hidden layer coupled with an additive step-wise approach for predicting colorectal cancer (CRC) using microRNAs (miRNAs). Three miRNAs were identified with a median accuracy 100% by using an extensive Monte Carlo cross-validation strategy. Sharma et al. [15] proposed a top-r feature selection technique that repeatedly divides and merge gene expression data to select the gene subset minimizing the loss of information. The selected genes are then tested on three tumor datasets and achieved higher accuracies than other feature selection methods, such as transcript-wise tests. Nanni et al. [46] examined the SVM classification performance using multiple feature reduction and data transformation approaches, including neighborhood preserving embedding, orthogonal wavelet coefficients, and texture descriptors. The study showed that a combination of different feature extraction methods could enhance genomic classification performance. For instance, the two combined methods achieved the highest average area under ROC curves (AUC) (AUC=92.18% for the WF method and 92.07% for the FUS method), while the AUC values for the 8 individual feature extraction methods were ranged from 79.24% to 91.85%. Jordan and Do [47] reviewed the studies that predict disease using full genomic information. Their review focused on polygenic risk scores (PRS), which is the most common method of integrating information from across the genome into a single estimate of genetic risk. A PRS is a weighted average of the genetic status at each associated risk locus. The weighting of each locus is usually the regression coefficient of genomewide association study (GWAS) association for the locus. Jordan and Do [47] mentioned that the power of most PRSs to predict disease risk has been very low due to several reasons, such as small sample size, genetic ancestry, heterogeneity of risk factors and causation.

The main limitations of these previous works [13] include (1) ignoring potential non-linear relationships among the features; (2) ignoring the contribution of features with weak signals to distinguish diseases; and (3) over-simplifying the complex prediction problem, such as using single-layer ANNs.

### Deep learning applications in disease prediction

Through a thorough literature search, we identified four papers [13,27–29] published between January 2013 and December 2017, which applied deep learning methods in disease prediction using genomic data (Table 1). The details of the four studies will be discussed below.

Fakoor et al. [13] is among the first to apply deep learning methods to extract key features from gene microarray data in predicting cancers. Fakoor et al. [13] compared three auto-encoders methods: a sparse auto-encoder with one hidden layer, a stacked auto-encoder with 2 hidden layers, and a stacked auto-encoder with fine-tuning. They first applied PCA to eliminate the effects of redundant and noisy dimensions, then applied the three auto-encoders methods to further extract non-linearly-correlated discriminating features based on the top principal components combined with some randomly selected original features, and finally used softmax regression to do classification based on the low-dimensional representations (Table 2). Thirteen gene microarray datasets were used to compare the performances of deep learning methods and two traditional prediction methods: Softmax based on the top principal components (PCA+Softmax) or SVM with Gaussian kernel based on the top principal components (PCA+SVM). The range of sample sizes of the 13 datasets is 20-1,047; the range of the numbers of features is 2,000-54,613. Ten-fold cross-validation was applied to estimate the average and standard deviation of the prediction accuracies and compared the average Accuracy (ACC) of the three deep-learning methods with the maximum of the accuracy of the two traditional methods. For 8 of the 13 genomic datasets, at least one of the three deep learning methods has significantly higher average accuracy than the maximum accuracy of PCA+Softmax and PCA+SVM. The median [min, max] increase of average ACC is 1.5% [0.7%, 8.3%]. The sample sizes of the 8 datasets range from 20 to 1,047. However, stacked auto-encoder without fine-tuning usually had much worse accuracy than the traditional methods. The stacked auto-encoder with fine-tuning achieved the best accuracy in six datasets with ACC ranging from 76.67% to 95.15%, while the single-layer sparse auto-encoder perform the best in 5 datasets with ACC ranging from 46.76% to 91.50%.

Tan et al. [27] used denoising auto-encoders to learn compact and efficient representations in predicting disease status. Tan et al. [27] used the Molecular Taxonomy of Breast Cancer International Consortium (METABRIC) cohort as the training set (1,424 samples) and the testing set (712 samples) and the cohort from The Cancer Genome Atlas (TCGA) as the independent evaluation set (547 samples). The DAE used in Tan et al. [27] has four layers: an input layer, a corrupted input layer, a hidden layer, and a reconstructed input layer. Each node in the hidden layer was used to predict disease status (e.g., tumor vs. non-tumor, or ER+ vs. ER−) depending on whether the node value for a sample in the evaluation set is greater than the optimal threshold that was obtained based on the discovery set and testing set. Tan et al. [27] showed that each of the top three hidden nodes in the discovery set could also have high prediction accuracy (>0.9) in the evaluation set when they used their method to predict tumor status (tumor sample vs. non-tumor sample).

Danaee et al. [28] used SDAE to transform high dimensional, noisy RNA-seq gene expression data to lower dimensional, meaningful representations, based on which they applied different machine learning methods to classify breast cancer samples from the healthy control samples. They also identified a set of “Deeply Connected Genes” (DCGs) that have strongly propagated influence on the reduced-dimension SDAE-encoding. Inspired by the classic study that applies SDAE to extract features in image data [40] Danaee et al. [28] built an SDAE model with four stacked layers of dimensions of 15,000, 10,000, 2,000, and 500, to obtain representations of genomic features to be fed into classifiers. An RNA-seq from TCGA is used to train and validate the model in the study. The dataset containsl,210 samples, including 1,097 breast cancer samples and 113 healthy samples. Danaee et al. [28] compared their prediction method with prediction methods based on PCA, Kernel PCA (KPCA, a non-linear PCA), the 206 differentially expressed genes (DIFFEXP0.05) that were significant at an FDR of 0.05 in gene-wised tests, and top 500 most significant differentially expressed genes (DIFFEXP500). Three classifiers, including a single-layer ANN, SVM, and SVM-RBF (SVM with a radial basis function kernel), were used to do the prediction based on extracted features. Like Tan et al. [27], Danaee et al. [28] used a training set and a testing set to train classifiers and used a validation set to evaluate the performance of the prediction methods. The classification result shows that the lowdimensional representations by SDAE outperformed other four sets of extracted features. For example, SDAE+SVM-RBF had accuracy (98.26%), sensitivity (97.61%), specificity (99.11%), precision (99.17%), and F-score [49] (0.983). Furthermore, Danaee et al. [28] showed that DCGs had slightly lower prediction accuracy than SDAE-extracted low-dimensional representations, but much higher prediction accuracy than the other methods.

Singh et al. [29] applied a stacked sparse auto-encoder (SSAE) to extract features to predict disease status for each of 36 datasets from the Gene Expression Machine Learning Repository (GEMLeR) [50]. The SSAE used by Singh et al. [29] has three hidden layers. The input layer contains top 800 features selected based on Individual Training Error Reduction (ITER) ranking. The three hidden layers have 700, 600, and 500 nodes, respectively. The three classifiers, Softmax Regression, kernel SVM, and Random Forest, were applied to the 500 extracted features to perform binary classification. Singh et al., [29] applied 10-cross-validation to estimate the classification accuracy and area under the ROC curve (AUC). Compared with the benchmark classification results taken from the GEMLeR website [50], the deep learning approach achieved slightly higher performance: ACC > 90.8% for 35 datasets (ACC>83.7% for all 36 datasets), and AUC>90.2% for 34 datasets (AUC >79.6 for all 36 datasets).

### Software packages for deep-learning-based feature extraction

Since deep learning algorithms usually are complicated, it is important to have open-source software packages available so that investigators can directly use these packages to their genomic data analysis. Both Tan et al. [27] and Danaee et al. [28] used *Theano* software that provides the implementation of auto-encoder algorithms. Fakoor et al. [13] and Singh et al. [29] did not mention the software packages that they used for auto-encoding.

Several software packages/libraries are available to build auto-encoder models and fine-tune model parameters. For example, *Scikit_learn, Theano, Keras*, and *TensorFlow* are Python packages. *h2o, kerasR*, and *autoencoder* are R packages. MATLAB has a Machine Learning Toolbox providing a set of functions for the easy implementation of deep learning methods. Wikipedia provides a table of deep learning software (https://en.wikipedia.org/wiki/Comparison_of_deep_learning_software).

## Discussion

In this article, we aimed to review all papers that applied the deep learning approach to predict disease status based on genomic data, which first obtains low-dimensional representations of high-dimensional genomic features, and then inputs these representations to the state-of-art classifiers that have excellent performance in lowdimensional classification problems. We found only 4 such papers, indicating that it is still in its infancy to predict disease status using deep learning on genomic data. However, the results of these 4 papers showed that the deep learning approach could extract useful genomic features from high-throughput whole genome data for prediction purpose with high accuracy.

Compared with commonly-used dimension-reduction methods, such as PCA and transcript-wise testing, the deep learning approach could have better performance in terms of a variety of accuracy measurements: ACC, AUC, sensitivity, specificity, precision, and F-score. Especially, it is impressive that transcript-wise testing, which is currently the most popular approach to identify disease-associated transcripts, performed poorly compared with PCA or auto-encoders [28]. However, whether the performance of the deep learning approach is significantly better than the commonly used approaches was not investigated in the 4 papers, among which only Fakoor et al. [13] provided standard errors for the estimated ACC. However, Fakoor et al. [13] did not provide some key details, such as the number of principal components used and the number of randomly selected raw features. They also did not provide p-values for testing if the mean ACC obtained using a deep learning approach is significantly better than that by using the PCA approach. Moreover, Fakoor et al. [13] showed that not all auto-encoders could outperform PCA. For example, Table 1 of Fakoor et al. [13] showed that for the first dataset, mean ACC (standard error) is 74.36% (0.062%) by using PCA+sparse auto-encoder, 51.35% (0.019%) by using PCA+stacked auto-encoder, while PCA approach had mean ACC 94.04% (SE 0.03%), although PCA+stacked auto-encoder with fine tuning (95.15% (0.047%)) performed better than PCA.

Different auto-encoders were used in the 4 papers, such as sparse auto-encoder, stacked auto-encoder, stacked auto-encoder with fine-tuning, denoising auto-encoder, stacked denoising auto-encoder, and stacked sparse auto-encoder. Except Fakoor et al. [13], the other three papers did not compare the auto-encoders used in the paper with other auto-encoders. Table 1 of Fakoor et al. [13] showed that PCA+stacked auto-encoder performed worse than PCA+sparse auto-encoder and PCA+stacked auto-encoder with fine-tuning in 12 of the 13 datasets. However, neither PCA+sparse auto-encoder nor PCA+stacked auto-encoder with fine-tuning could outperform each other in all 13 datasets. For a fair comparison, it could be beneficial for future studies to compare the deep learning methods mentioned above using the same datasets.

All four papers mentioned the number of hidden layers and the number of nodes in each hidden layer used for the auto-encoders. However, no justifications and guidance were given on why choosing those specific numbers of hidden layers and those specific numbers of nodes in each hidden layer. This is probably one of the main reasons why deep learning has not been widely used in the genomic research area. There are some existing methods to choose the number of layers and nodes, such as (1) starting from a small neural network and adding layers and nodes until the error stops decreasing, and (2) starting from a big neural network and remove layer and nodes until the error increases significantly [51]. Optimization methods such as grid search and random search are also proposed and discussed [52] to optimize the parameters in model training. However, these methods are still not well studied in genomic data analysis and could not eliminate the risks of over-fitting and under-fitting. Future research is still needed in choosing and optimizing deep learning parameters, especially in genomic data analysis.

Another possible reason why deep learning has not been widely used in the genomic research area is the lack of software packages that implement deep learning algorithms for genomic data analysis. Many investigators in genomic research area use the R language and use packages in Bioconductor, a repository of R packages specifically for genomic data analysis. Although there are a couple of R packages, such as *keras* and *kerasR*, connecting R to the Keras deep learning library, there is lack of examples and tutorials on how to use them to analyze genomic data and to visualize the low-dimensional representations that are obtained by auto-encoders.

It is a non-trivial task to interpret the low-dimensional representations (features) of the original expression data obtained by auto-encoders because the representations are non-linear functions of gene transcripts and the hidden layers in deep learning algorithms are like “black box” [53]. Among the 4 papers that we reviewed, Tan et al. [27] and Danaee et al. [28] suggested interpreting the representations based on the transcripts having strongly propagated influence on the reduced-dimension auto-encoding. However, no details were given on how to select these transcripts, except that these transcripts have high edge weights.

To evaluate classification performance, several measurements were used in the four papers that we reviewed, including accuracy (ACC), area under the ROC curve (AUC), sensitivity, specificity, precision, and F-measure. We call a dataset is imbalanced if the number of cases/positive samples is much different from that of controls/negative samples. When the dataset is imbalanced, using ACC could be biased. For example, given a dataset with 99% true negative samples and 1% true positive samples, a classifier could achieve 99% ACC even if it wrongly classifies all the true positive samples to the negative group. Fakoor et al. [13] only used ACC as the performance metric, while several genomic datasets analyzed in Fakoor et al. [13] are imbalanced. Tan et al. [27] also only used ACC to evaluate the performances of different prediction methods, while both the training and testing datasets are highly imbalanced. For imbalanced data, other performance metrics can be used, such as AUC, F-measure, and G-measure [49,54], which are less sensitive to the case/control imbalance.

Over-fitting is a big issue in prediction. Using the same data set to both train the prediction model and evaluate the performance of the prediction model usually causes over-estimation of the prediction accuracy. Ideally, a testing set from a population independent of the training population is required in evaluating prediction accuracy. However, genomic data are usually expensive to collect. Hence, it is usually hard to obtain independent testing set in genomic research. Thanks to the policy of the National Institute of Health of the United States, numerous genomic datasets are now publicly available in the Gene Expression Omnibus (https://www.ncbi.nlm.nih.gov/geo/), an online repository of genomic datasets. Other public genomic repositories are also available, such as TCGA (https://cancergenome.nih.gov) and GTEx (https://www.gtexportal.org/home/). Hence, nowadays it is relatively easy to obtain an independent testing set for most complex human diseases. Among the 4 papers that we reviewed, only Tan et al. [27] used an independent testing set. The other 3 papers used K-fold cross-validation technique to alleviate the over-fitting issue.

Genomic data usually contain many sources of technical noise, such as batch effects due to that large samples have to be handled in multiple batches due to capacity limits of machines. Several methods, such as ComBat [55], have been proposed to remove the effects of technical batches before downstream data analysis. We can apply ComBat to the training set and the testing set, separately. Suppose after removing technical noises we build and validate a prediction model based on the training set and the testing set, with excellent prediction accuracy. Now a new subject’s genomic data are obtained. Can we apply the prediction model to this new subject? The answer probably is “no”, since we do not know how to remove technical noises for only one new sample. One possible solution is to collect genomic data for a batch of subjects together. Then we can apply the prediction model to subjects in this batch after removing possible batch effects. A possibly better solution is to improve technology to reduce technical noises. With the advancements in sequencing technology and a rapid decline in sequencing costs, DNA sequencing has gained remarkable popularity among biomedical researchers. Compared to microarrays, DNA sequencing data is believed to deliver faster, more complete, and more scientifically accurate genomic analysis [56].

The four deep-learning papers identified in this review compared the performances of deep learning approaches with PCA approach and transcript-wise test approach. There are many more advanced feature selection methods in the literature, such as the stable feature selection method [16] and the Boruta algorithm [17]. More comprehensive comparisons are warranted.

Recently, the authors [29] improved their results using deep transfer learning [57]. Moreover, semi-supervised learning and reinforcement learning are receiving a lot of attention to image recognition, gaming, and robotics [58–60]. How to apply the frontier deep learning innovations to genomic data analysis could be an interesting future research topic [61].

Finally, we would like to mention a few related review articles on deep learning. All these reviews are pretty broad and do not focus on prediction of disease status using genomic data. Ching et al. [61] examined applications of deep learning to a variety of biomedical problems, including patient classification. They only briefly mentioned disease prediction based on autoencoders and cited Tan et al. [27]. Miotto et al. [62] reviewed the application of deep learning in the healthcare domain and cited Fakoor et al. [13]. Mamoshina et al. [63] reviewed the application of deep learning in biomedicine. Angermueller et al. [64] reviewed the application of deep learning in regulatory genomics and cellular imaging. To the best of our knowledge, our review is the first focusing on the prediction of disease status based on deep learning, which is an important component in personalized medicine.

## Conclusion

In summary, this review showed that applying deep learning to find a low-dimensional representation for high-throughput genomic data is a promising future trend in disease prediction based on high-dimensional genomic data. The low-dimensional representation obtained by deep learning could capture both linear and non-linear relationship among the transcripts. Deep learning is a new technology for most scientists in genetics. Scientists in genetics should collaborate to understand how deep learning could help predict disease status using genomic data, hence to move this field forward.

## Figures and Tables

**Table 1:** Summary of the four studies that applied deep learning to predict disease status in the genomic research.

Author:Year	Training			Testing			Method	Methods compared	classifier	CV	Performance
	Type	N	P	Type	N	p					
Fakoor [13]	13 array-type datasets	20-1047	2000-54675	NA	NA	NA	PCA+SpAE; PCA+StAE; PCA+StAEf	PCA	(1) softmax regression; (2) SVM with Gaussian kernel	10-fold	ACC ± SE: (33.7% ± 0.038%)-(97.5% ± 0.079%)
Tan [27]	METABRIC array type	2136	2520	TCGA array type	547	2520	DAE	NA	Sigmoid activation	10-fold	ACC: 75%-99.6%
Danaee [28]	TCGA RNAseq	1210	NA	NA	NA	NA	StDAE	PCA; KPCA; DE	ANN; SVM; SVM-RBF	5-fold	ACC: 96.95%-98.26; Sen: 97.21%-98.73%; Spec: 95.29%-99.11%; Prec: 95.42%-99.17%; F-measure: 0.970-0.983
Singh et al.[29]	36 array type datasets from GEMLeR	1545	54676	NA	NA	NA	SSAE	KNN; SVM-RFE	Softmax regression; random forest; linear SVM; SVM-RBF	10-fold	AUC: 80%-100%; ACC: 76%-100%

N: number of samples; p: number of features; CV: cross-validation; SpAE: sparse auto-encoder; StAE: stacked auto-encoder; StAEf: stacked auto-encoder with fine-tuning; DAE: denoising auto-encoder; SDAE: stacked denoising auto-encoder; SSAE: stacked denoising auto-encoder; DE: differential expression analysis; ACC: accuracy; SE: standard error; AUC: area under ROC curve; Sen: sensitivity; Spec: specificity; NA: missing

**Table 2:** A summary of different auto-encoders.

Method	Description
Regular auto-encoder (AE)	Find low-dimensional representation of input using an unsupervised approach (i.e., no outcome information is used)
Sparse AE (SpAE)	Restrict the number of hidden nodes to be activated to avoid too many free nodes with complex dependencies in each layer
Stacked AE (StAE)	Each hidden layer is a low-dimensional representation of the previous layer obtained by AE
Denoising AE (DAE)	Introduce noises to input to make AE more robust to noises
Stacked denoising AE (SDAE)	Combine stacked AE and DAE (i.e., introduce noises to input in a stacked AE)
Stacked sparse AE (SSAE)	Combine stacked AE and SpAE (i.e., introduce sparse restriction on the stacked AE hidden layers).
